# Embedding Technological Tools Into Screening for Family Violence in Antenatal Care: Views of Australian Midwives

**DOI:** 10.1177/10778012251401898

**Published:** 2025-12-12

**Authors:** Minerva Kyei-Nimakoh, Caroline Bert, Kelsey Hegarty

**Affiliations:** 1 Faculty of Medicine, Dentistry and Health Sciences, Department of General Practice, 85084 The University of Melbourne, Melbourne, VIC, Australia; 2 Centre for Family Violence Prevention, 2282 The Royal Women's Hospital, Parkville, VIC, Australia

**Keywords:** family violence, antenatal care, screening, technological tools, midwives

## Abstract

The prevalence and adverse health consequences of family violence among pregnant women highlight a need to develop effective screening tools in antenatal care. We explored how antenatal care midwives at a tertiary maternity hospital in Melbourne perceive the use of technological screening tools for family violence. Seven face-to-face and 10 telephone interviews were conducted. Data analysis was conducted using thematic analysis. Five themes were identified: clinician readiness for family violence screening; challenges of existing screening protocol; enhancing processes with technology; fostering disclosure and help-seeking; and coupling technology with face-to-face discussion. Overall, midwives were receptive to computerized family violence screening modalities.

## Introduction

Intimate partner violence (IPV) is behavior by an intimate partner or ex-partner that causes physical, sexual, emotional, or financial harm (World Health Organisation, 2017). Family violence (FV) is a broader term, encapsulating violence between family members, as well as intimate partners, or ex-partners (Phillips & Vandenbroek, 2014). Pregnancy represents a period of heightened risk for the onset or worsening of IPV (Australian Institute of Health and Welfare [AIHW], 2015). Adverse pregnancy outcomes such as premature labor, low infant birth weight, and neonatal death are among the detrimental health outcomes associated with IPV (Sarkar, 2009). Embedding evidence-based, effective IPV screening into the antenatal care system aims to identify women experiencing IPV, who may not be in contact with IPV services (O’Doherty et al., 2014). As almost all women in Australia have ongoing contact with the health system during their pregnancy, IPV screening during antenatal care represents a valuable opportunity for intervention (AIHW, 2015). For this reason, national clinical practice guidelines for antenatal care now recommend that midwives routinely enquire about each woman's exposure to IPV at the first antenatal visit (Australian Health Ministers’ Advisory Council, 2012).

Despite this, barriers to effective IPV screening exist (Sprague et al., 2012). A lack of IPV knowledge, time constraints, language barriers for culturally and linguistically diverse (CALD) populations, and a lack of confidence in management of IPV disclosure have been emphasized among health professionals as barriers to effective screening (LoGiudice, 2015; Sprague et al., 2012). In Australia, this lack of confidence in management of IPV disclosure may be attributed to an inconsistency in the provision of education and training programs for midwives in the area of IPV enquiry (Baird et al., 2015). A survey of Australian midwives revealed that most had received some training in IPV enquiry; however, this varied from attending a lecture about IPV, to reading hospital policy, or attending a skill-based IPV workshop (Baird et al., 2015).

Technological screening tools may contribute to overcoming barriers to effective IPV screening (Renker, 2008). Comparison of IPV screening modalities in a systematic review and meta-analysis of six randomized controlled trials found computer-assisted, self-completed screening modalities may lead to higher rates of IPV disclosure in comparison to both face-to-face interview and self-completed written modalities (Hussain et al., 2015). Furthermore, women seem receptive to computerized IPV screening (Chang et al., 2012; Choo et al., 2015; Tarzia et al., 2018). In a hospital-based antenatal clinic, computerized, self-completed screening was perceived by women as more anonymous in comparison to face-to-face screening, allowing disclosure without fear of judgment (Chang et al., 2012). In an Australian study of women who had experienced IPV, women felt that many elements of face-to-face support from a health professional could be delivered effectively using technology but noted that reassurance and a trusting relationship with a health professional may be difficult to replicate online (Tarzia et al., 2018).

The acceptability of computerized modalities for IPV screening has also been emphasized among health professionals (Alvarez et al., 2018; Bacchus et al., 2016; Decker et al., 2017; Hawkins et al., 2009; Rickert et al., 2009; Scribano et al., 2011), with limitations of paper-based IPV screening providing the rationale for embedding computerized screening in some settings (Hawkins et al., 2009; Scribano et al., 2011). Despite the literature demonstrating an understanding of how health professionals perceive computerized modalities for IPV screening, an appraisal of the evidence highlights a lack of research specifically addressing the views of midwives. This gap in current knowledge regarding how midwives perceive the use of technological screening tools is particularly important to address given a move towards screening for IPV in antenatal care settings (O’Doherty et al., 2014). This study therefore aims to provide novel insight into how Australian antenatal care midwives perceive computerized screening modalities for IPV, and more broadly FV.

## Method

### Study Context

The study was undertaken at a tertiary maternity hospital in Melbourne. In April 2019, antenatal care midwives in one unit piloted routine paper-based FV screening involving the woman filling out a form at her first antenatal appointment. The hospital then introduced routine, mandatory paper-based FV screening across all units of antenatal care midwives which was done during the woman's first antenatal appointment. Once privacy has been established, the midwife asks the woman a series of questions based on a validated FV screening tool (Hegarty et al., 2021), addressing her home life and relationships.

*In the last year, has a partner, ex-partner or other family member:*

**Table:** 

*Done something that made you feel afraid?*	□ *Yes*	□ *No*
*Controlled your day-to-day activities (e.g., who you see, where you go) or put you down?*	□ *Yes*	□ *No*
*Threatened to hurt you in any way?*	□ *Yes*	□ *No*
*Hit, slapped, kicked or otherwise physically hurt you?*	□ *Yes*	□ *No*

If a woman answers ‘yes’ to any of the above questions, an individual safety and needs assessment is initiated. At the time of the study, the hospital had plans to introduce an electronic medical record (EMR) screening protocol to replace paper-based systems.

### Study Design and Sample

An exploratory qualitative approach was used to explore how midwives perceive the use of technological screening tools for family violence. Between April and August of 2019, face-to-face and telephone semi-structured interviews were conducted with a convenience sample of 17 midwives who provide antenatal care. All antenatal care midwives at the hospital were eligible to participate in the study. Recruitment of midwives took place through hospital networks, via differing methods, including the provision of Expression of Interest forms at two midwifery team meetings and snowball sampling within midwifery staff networks. No incentives were offered for participation; however, findings were made available to participants in de-identified form.

### Data Collection

Seven face-to-face interviews were undertaken in private spaces at the hospital, and ten telephone interviews were conducted from a private meeting room at a university. Prior to their scheduled interviews, participants were asked to complete a demographic survey. An interview guide was used to facilitate discussion, and consisted of a series of non-leading, open-ended questions addressing current screening practices, how technology may affect screening efficiency, comfort in discussing FV, and potential advantages and issues midwives may foresee regarding computerized FV screening. Midwives were first asked about their relatively new paper-based screening practices for FV during antenatal care, followed by a discussion of their perspectives on technology when that becomes available through EMR, e.g., having the woman complete the screening tool on an iPad, instead of a paper form.

Interviews were audio recorded on two devices and had an average duration of 25 minutes. Non-identifying verbatim interview transcripts were produced from the interview recordings, and coding of file names ensured potentially identifying information was removed. Hand-written field notes made during the interviews supplemented audio recordings and allowed documentation of general thoughts and observations.

The interviewing researcher was an honors’ student undertaking the study as part of their honors program. She worked closely with two supervisors during this period and was provided with guidance and support throughout the project cycle. The interviewer commenced this project with few preconceptions related to the research topic. However, this study was rooted in the context of technology playing an increasingly important role in the evolution of healthcare with the introduction of EMR in the months following the study period. Therefore, using reflexivity (Jootun et al., 2009) and introspection to recognize any preconceptions regarding the value of technology in healthcare, and approaching the research with an open mind, were essential in increasing the credibility of the research process.

### Data Analysis

Data analysis was concurrent with data collection. Thematic analysis involved descriptive coding of transcripts, categorization of codes, and identification of themes (Braun & Clarke, 2006). Continued reflection on the transcripts allowed refining of the interview guide. The development of the coding framework was achieved through a process of deliberation among the research team members. The coding process involved critical engagement with the nuances and complexities in the data, acknowledging the researchers’ positionality and the contextual nature of the interpretive process. We coded the data with handles that identified key concepts or reflections in an iterative manner, and an audit trail of code generation contributed to the rigor of the data analysis. A combination of deductive and inductive approaches meant some themes were identified in advance, and others were identified from deeper exploration and interrogation of the data. NVivo 12 software (QSR International Pty Ltd, 2018) assisted with data coding and the development of a thematic framework. All authors reviewed and validated the coding, and identified themes were discussed to reach a consensus, minimize individual bias, and ensure validity (Barbour, 2001). The final thematic framework and selection of illustrative quotes were discussed collaboratively in an effort to ensure credibility. In order to establish trustworthiness, techniques such as reflexive journaling, diagramming to make sense of theme connections, and researcher triangulation were used to ensure transferability, dependability, and confirmability (Lincoln & Guba, 1985).

### Ethical Considerations

Ethical risks posed by this research were minimal and verbal informed consent was obtained from all participants. A distress protocol was developed to respond to any risk of evoking emotional distress. Ethics approval was obtained as a quality assurance activity, as part of a broader study, The SUSTAIN Study (Ethics ID: Project 17/35), in accordance with the National Health and Medical Research Council (NHMRC) guidelines (NHMRC, 2014). To ensure transparency, reporting followed the Consolidated criteria for Reporting Qualitative Research guidelines (Supplementary File 1) (Tong et al., 2007).

## Results

### Participant Demographic Background

Seventeen antenatal care midwives working at the maternity hospital participated. Most were female (*n* *=* 16), with the median age (years) falling in the 30–39 category*.* Most midwives (*n* = 14) had experience using the screening tool. Despite diversity in years of practice, all had at least six months of experience as a midwife and 7 (41%) had between 5 and 9 years of experience. Overall, 13 midwives had received at least one hour of FV training, 6 (46%) had 1–5 h training and another 6 (46%) had received 10+ hours of FV training. Four midwives indicated they had not had any FV training (Table 1). Of the midwives with FV training, some had completed online modules which aimed to improve the confidence and competency of health professionals in identifying and responding to FV, while others had received more in-depth, face-to-face training.

**Table 1.** Participants’ Demographic Characteristics.

Participant code	Years at health service	Years^a^ as midwife in antenatal care	FV training	Hours of FV training	Age	Sex
01	4	7	Y	20 h	40–49	Female
02	19	19	Y	3 h + full day	50–59	Female
03	8	9	Y	1 h	40–49	Female
04	7	15	Y	3 h	40–49	Female
05	1	1	N	N/A	40–49	Female
06	4.5	5.5	Y	1 h	20–29	Female
07	0.5	0.5	Y	3.5 h + training at university	30–39	Male
08	1.5	1.5	Y	3.5 h + training at university	20–29	Female
09	1.5	5	Y	5–10 h		Female
10	10	2 weeks	Y	3 h	40–49	Female
11	10	4	Y	1 h	50–59	Female
12	2	5	N	N/A	20–29	Female
13	5	3	Y	3.5 h + training at university	20–29	Female
14	5	11	N	N/A	30–39	Female
15	4	4	N	N/A	20–29	Female
16	6	8	Y	2 h	40–49	Female
17	5	5	Y	10–15 h	30–39	Female

^a^Column content in years except otherwise indicated (as for participant 10).

### Themes From the Interviews

Five themes were identified from the interviews, clinician readiness for FV screening; challenges of existing screening protocol; enhancing processes with technology; fostering disclosure and help-seeking; and coupling technology with face-to-face discussion.

#### Clinician Readiness for FV Screening

Considering family violence screening had newly been introduced at the hospital at the time of the study, some midwives described feeling ill-equipped to deal with a positive disclosure.

Sometimes I’ve got no idea. I wouldn’t know quite what to say apart from call the police. (14)

It's always tricky, I’m always conscious of what I’m going to be asked next, and will I give them the right information? Which … I think it's still lacking, and I know the hospital is pretty aware of it, but our lack of knowledge about how to answer some questions is present. (06)

The sense of unpreparedness to provide appropriate support and education after a disclosure was fundamentally linked with a lack of FV training, and because screening for FV was new to them. Most midwives wanted more specific training regarding how to phrase questions and respond to a disclosure. Some midwives described a lack of knowledge about available resources and services, and what to say when a woman discloses. One midwife highlighted the value of face-to-face education in making her feel more comfortable with screening.

I think if more people did that [FV training], they wouldn’t be so freaked out about the idea that someone might disclose family violence, and I don’t know what I’m going to say if they do. (17)

Despite screening for FV being difficult at first, most midwives felt that developing their own way of phrasing things helped them become more comfortable with introducing the topic of FV.

#### Challenges of Existing Screening Protocol

All midwives perceived screening for FV during antenatal care as important, however, midwives described additional challenges encountered while employing the paper-based processes in place.

The screening tool interrupted the flow of the antenatal appointment for some midwives.

It's been nice to have a bit more guidance in terms of what questions we should ask women, but it still feels a bit clunky. (13)

Us having to copy in all the stuff and chase all the tests that the GPs haven’t sent in, and ask them how they’re feeling, and talk about what their options are at the hospital and get the partner in the waiting room out of the waiting room … this has all been added in with no extra time for the appointment. So, it just sort of means that you have to save time somewhere else, but we don’t really have anywhere to save it … So, it’d be nice if we could find a more efficient way to do it. (13)

In addition, when reflecting on their current practice, most midwives noted a lack of rescreening of women in subsequent appointments.

I feel like we do the screening, and it all seems okay, and then we file it away and it's not revisited. I think maybe we should be asking again later in pregnancy. Like we do with mental health. (02)

In addition to the fact that paper forms are filed away or buried in other notes, several midwives attributed this lack of rescreening to the time barrier in review appointments, and the absence of a policy in place around revisiting the screening tool.

Most midwives revealed challenges with screening CALD women as the screening tool was not available in other languages. Language barriers represented important hurdles, and several midwives described telephone interpreting services as problematic, particularly given there was not always a female interpreter available, and at times the call would cut out. Furthermore, some midwives expressed concern that women may not feel comfortable disclosing in the presence of an interpreter. Midwives revealed divergent understandings of whether, and how, non-English speaking women should be screened.

I think the language barrier is for sure a huge thing at the moment, where it's stopping the women from being screened. And in my experience, they’re some of the ones that are most at risk. (08)

Despite acknowledging these issues, most midwives, nevertheless, regarded the screening tool as valuable.

I feel like this [screening tool] has given us a chance to delve into things and I’ve definitely made more referrals than I would have ever made before this screening tool has come out. (08)

#### Enhancing Processes with Technology

Technology use was an accepted norm in clinical practice and network errors, or system glitches were not important concerns. Despite diversity in age, most midwives were comfortable with using technology in appointments, as well as having the woman use technology.

I think most people are okay with using technology, and probably expect to use it, more so than paper-based. (02)

Furthermore, most midwives did not perceive technology as a barrier to communication, predicting that regardless of modality, the woman will disengage from the midwife and engage with the screening tool.

I suppose people do tend to tune out with their phones and stuff. Whether that – I don’t know if that would happen. Hmm. I think it’d be the same thing giving someone an iPad as it would be giving them a form to fill out. They still have to disengage from you and engage with the form or iPad. I wouldn’t want the whole booking visit to be done online or on an iPad … I think the face-to-face element is really important. But I think a little bit of technology would be good and helpful. (14)

For some midwives, reflecting on their current FV screening practice and its limitations led to ideas of how technology could assist the screening process in important ways. Technology was perceived as potentially enhancing efficiency, particularly in the context of the hospital moving to EMR.

I’m not sure if, when EMR comes out we’ll still give women the paper form and we’ll have to enter it into the computer. Because if so, that would be annoying and time-consuming … It seems like a natural progression that family violence screening becomes electronic. (17)

In addition, one midwife shared how technology could be useful in highlighting ‘red flags’ in the woman's file.

Say there are some red flags for domestic violence, that's not always really bold in their history so those kinds of things could get missed, whereas if it was all electronic, they might be easier to find and highlight. Because with the paper history there's just so much in there that I do sometimes feel like things get missed. (09)

Some midwives felt technology may be valuable in translating the screening tool into the woman's language.

If it could be interpreted into that woman's language that would be amazing and probably better than what we’ve got now for women who don’t speak English. (16)

In addition to enhancing efficiency, midwives felt that technology could guide midwives during FV screening in ways which paper-based screening modalities could not. Most midwives felt that guidance, in terms of available resources, things to say, or strategies to suggest depending on the woman's current circumstances, would help them to feel well-supported in their work in screening.

If they say “yes” to this question, then you could say, “these are a couple of things you could say”, or “these are a couple of things you could ask them about”. Not necessarily completely putting the words into our mouth but just offering some options of what exactly to say and how to phrase it. Because it's an awkward subject matter. (14)

One midwife reflected on the benefit of having tools or resources in front of them to give the woman in the interim, while she is waiting for social work to contact her, or if she declines a referral.

If there was a link for the midwife, you know, if she's answered “yes” to this question, being it physical or whatever, these are our resources of where you can refer to … But if you’ve got, okay it's this happening, we’ve got links to the Centre Against Sexual Assault … it would be helpful if there was a pathway. (16)

Another midwife discussed the utility of having a policy in place regarding what to discuss with the woman.

We live by policy. We live by flowcharts of, if this happens, do this. I think it’d be fantastic to be able to have a resource of, if they answer “yes” to Question 10, the automatic policy procedure is to do this, or to have this discussion. (12)

#### Fostering Disclosure and Help-seeking

The idea that technology could foster disclosure and promote help-seeking behaviors was a strong theme among midwives. Some midwives predicted that technological screening tools may support women in feeling more comfortable disclosing, particularly as screening for FV takes place on the woman's first antenatal appointment, when a rapport may not yet be established.

We are meeting the woman for the first time, and getting that rapport is difficult … I guess [computerized screening] gives them the opportunity to disclose without having to do face-to-face which might be awkward for the first appointment. (11)

[Computerized screening] might be a little less confronting … It could take out some of the challenging interpersonal feelings. (04)

Furthermore, a key advantage of computerized screening was identified as the potential to embed tailored educational messages which may encourage women to reflect and self-assess and perhaps be able to open up to the midwife in a subsequent appointment.

I think [education] is the first step, and then face-to-face is really important, because sometimes people don’t disclose straight away. So maybe if you get a little bit of education, and then maybe the next time they see the midwife they might open up when before they weren’t able to. (11)

One midwife emphasized that technology could allow for immediate display of resources after a positive screen, proposing this could open up more avenues for the woman.

It would be handy to be able to immediately show them where they might find resources online … which could, I suppose, open their mind to what it's like, and that it's not a really hard process to start that ball rolling … I think a visual prompt, to be able to say, “if you go to this website” … is a good thing for us to be able to use as midwives, given our limited knowledge on that front, to be able to say, “we care about you”, “we can help you immediately, but you can also look at this kind of stuff and get some help yourself”. (06)

She then reflected on the utility of displaying resources on the screen for the woman to look at while the midwife writes a referral.

We could be writing a referral while they start to look around – because I’d imagine one element that could be a reason they hadn’t sought help is because they don’t know how to easily access information … It’d just be utilizing that little bit of down-time briefly to say, “have a look at these resources” while we write a couple of things, and then you can continue with the discussion … Because they’re just sort of waiting and watching which often just feels horrible if it's a situation where they’ve disclosed something quite serious, and you sort of wish there was more that they could be doing while you’re just writing a few bits and pieces. (06)

#### Coupling Technology With Face-to-face Discussion

Given the important advantages technology could deliver in screening for FV, the coupling of technology with face-to-face discussion developed as a strong theme and it was largely seen as a positive way forward. Almost all midwives revealed a strong preference towards embedding technological screening tools while maintaining a face-to-face element and they viewed the pairing of these screening approaches as complementary.

There's nothing like having a face-to-face conversation. That's going to be the most powerful thing. (08)

Face-to-face discussion allowed midwives to probe women to ensure they understood questions, interpret non-verbal cues, and acknowledge a disclosure and offer support and reassurance.

You can ascertain how she reacts and that does really impact on your referral … if she's looking closed up and not wanting to engage that rings alarm bells and you can include it in your referrals. (15)

If someone has said yes to a health service in some capacity, then it's probably important to acknowledge that they’ve said something and ask what support they’d like at that time. (13)

Despite the majority of midwives feeling more comfortable providing support and education about FV in a face-to-face context, they acknowledged that some women may be more comfortable disclosing FV and receiving information electronically.

It's possible that some people would be so reluctant to go into any depth about what's going on in a face-to-face conversation, and might be more willing to write down more detail on a device as it's less sort of threatening than having that conversation … But at the same time, I think if someone is willing to talk, having somebody who's emphasizing and listening, and able to tailor that conversation to what they need to be talking about would be better than having a device. (04)

However, several midwives felt a reliance on technology may impact negatively on the interpersonal element of midwifery.

I think having an iPad is almost like having a … for me like, a barrier between the woman and the midwife. (01)

I don’t want technology to get in the way … I feel like sometimes we’re sort of losing that human contact, which I think is really important in midwifery. We don’t want too much computer work, otherwise it just becomes us talking to the computer. (11)

One midwife expressed concern that a glitch, such as a network connection error, may depersonalize the experience of disclosing.

Generally, it's fine. You can log out and log in again … But that might, I suppose, depersonalize the experience, if you’re just about to disclose something and then there's no network connection … That glitch – you’ve interrupted that momentum of somebody potentially disclosing. (16)

Figure 1 shows how the coding process led to identification of the five main themes that capture key findings. Each branch consists of supporting details to further elaborate on the relationships between themes and sub-themes, highlighting the nuanced perceptions of midwives. It shows the ongoing tension between the benefits and challenges of implementing computer-based family violence screening tools in clinical practice. Overall, it indicates that while computer-based screening tools offer several advantages, significant concerns, user experiences, and other issues arising must be acknowledged and addressed.

**Figure 1.**
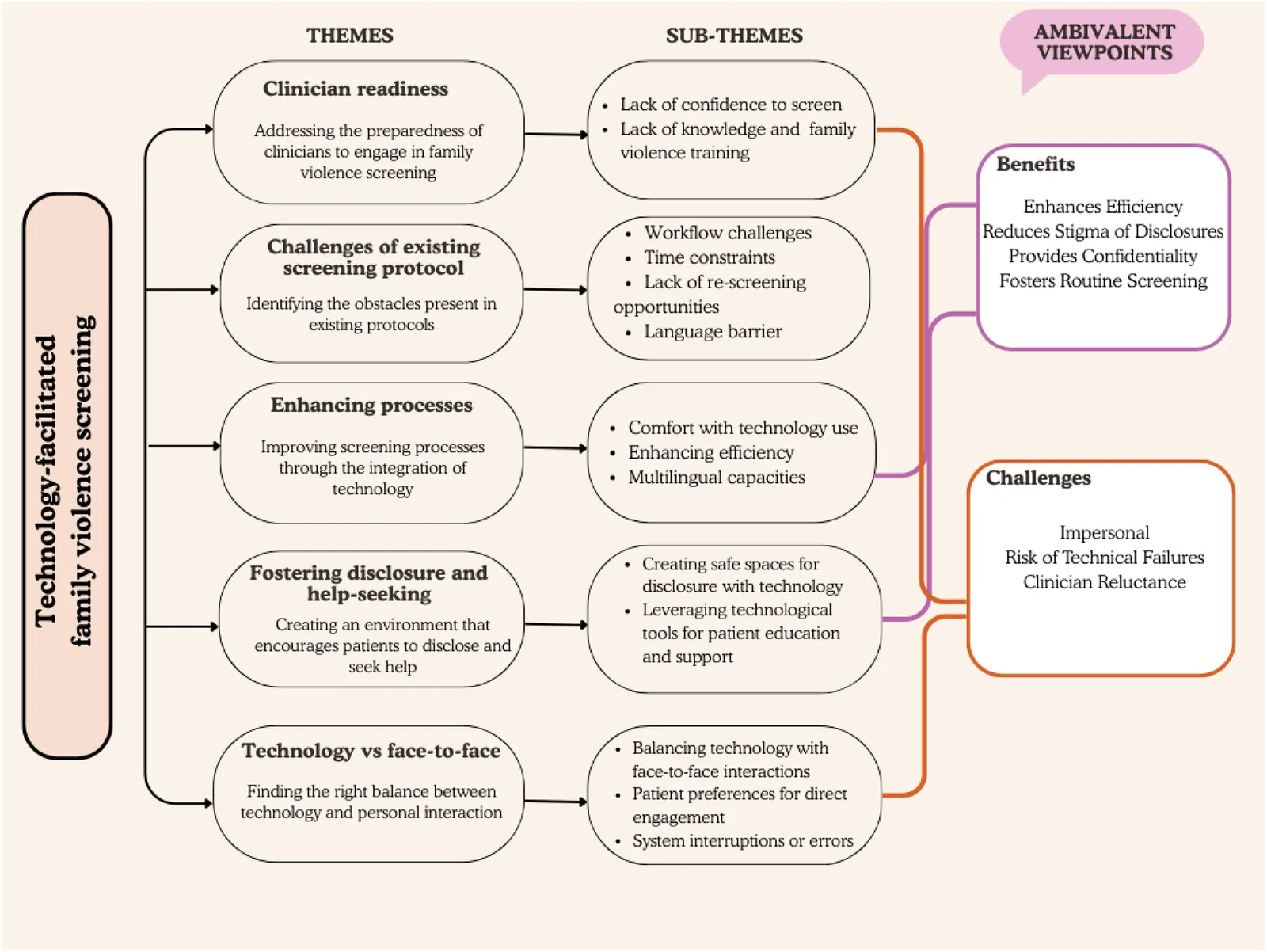
Themes, Sub-Themes, and Midwives’ Ambivalence Surrounding Technological Tools in Healthcare Practice.

## Discussion

### Overview of Findings

In this study we aimed to explore how midwives perceive the use of technology in screening for FV during antenatal care. The study was undertaken in the context of introduction of a relatively new paper-based FV screening protocol at the site. Consistent with existing findings on health professionals (Alvarez et al., 2018; Bacchus et al., 2016; Decker et al., 2017; Hawkins et al., 2009; Rickert et al., 2009; Scribano et al., 2011), midwives were generally receptive to computerized FV screening, highlighting the utility of technology in overcoming barriers of paper-based screening, assisting processes, and fostering self-reflection among women. Some midwives shared that they felt ill-equipped to provide support and education following a disclosure, which for some, provided the rationale for adopting a computerized screening modality where guidance in responding to a disclosure could be embedded. While most midwives felt that using technology could complement and enhance their work in FV screening, the importance of maintaining a face-to-face element was emphasized. Some midwives expressed discomfort in asking sensitive FV screening questions with some suggesting that developing their own way of phrasing questions may ease their discomfort with introducing the topic of FV. A study by Rollans et al. (2013), indicates that modification of questions by clinicians may impact on the integrity of the assessment tool as they sometimes stray away from the well-tested and evidence-based screening questions. A computerized FV screening system would help alleviate this concern.

Consistent with the present study, in a qualitative meta-synthesis exploring health professionals’ experience with paper-based antenatal IPV screening, midwives felt inadequately prepared and ill-equipped to respond to a positive disclosure (LoGiudice, 2015). Recent Australian-based research attributes this unpreparedness to handle a disclosure to IPV-related content being poorly embedded in the midwifery undergraduate curriculum, and attitudes among midwives that IPV-related care is beyond the scope of their practice (Lovi et al., 2018). Similarly, in the present study, midwives acknowledged a lack of FV-specific training and education, but, in contrast to Lovi et al. (2018), displayed a positive attitude towards FV screening and emphasized a desire to be better-equipped to screen. Given the IPV-related training Australian midwives receive is inconsistent, and at times lacking, there is a need for continued provision of robust, on-going training in the area of IPV enquiry, so that midwives feel sufficiently prepared in their work in screening (Baird et al., 2015). To help embed routine FV practice, domestic violence champions have been recognized as playing an important role in mentoring and supporting clinicians (Saberi et al., 2022). However, findings from this study indicate the implementation of technological screening tools could also contribute to midwives feeling more supported, give them something to fall back onto, and help build their confidence in managing a disclosure. Therefore, on-going education and training in IPV enquiry, coupled with the guidance that technological screening tools could offer, may assist and empower midwives in their future work in screening.

Similar to earlier findings (Alvarez et al., 2018; Hawkins et al., 2009; Scribano et al., 2011), technology, if implemented appropriately, was perceived as a valuable tool in overcoming limitations of paper-based screening, and in assisting processes. Existing research attributes this enhanced workflow efficiency to a reduction in redundant data collection and the facilitation of coordination of support services (Alvarez et al., 2018; Hawkins et al., 2009; Scribano et al., 2011). Midwives in this study attributed an improvement in efficiency with a computerized modality to its potential to interface well with the EMR, which had not been discussed in previous literature (Alvarez et al., 2018; Hawkins et al., 2009; Scribano et al., 2011), but is an important consideration given health professionals are increasingly engaged in a rapid transition from paper-based to electronic patient records (Cáceres, 2013). A computer-based system would also allow screening records to be kept in a manner that keeps FV screening in view rather than buried in a filing system. Potentially, this will also allow instituting flags in the EMR for rescreening or follow-up.

In the present study, midwives felt it would be useful if guidance could be embedded in a computerized screening modality. Having immediate access to resources, things to say, strategies to suggest, or other tools to offer the woman, particularly if she was to decline a social work referral, was perceived as a key advantage of computerized FV screening. The responses suggest that midwives would potentially benefit from a form of algorithm or decision-making aid that may guide their practice. This represents a novel finding, as the concept of utilizing technological screening tools for FV to guide health professionals’ management of a disclosure has received little attention to date.

Similar to other research (Alvarez et al., 2018; Bacchus et al., 2016), midwives in this study felt that providing education about FV to all pregnant women could be enhanced through computerized screening modalities, which could allow for interactive features and flexibility in tailoring intervention content to women's changing needs and priorities. Another way in which computerized screening was perceived to foster disclosure was related to how this modality could allow women to receive information, or self-refer, without having to discuss their situation in a face-to-face conversation. This is in line with established literature which suggests women are receptive to computerized screening and advice (Chang et al., 2012; Choo et al., 2015; Tarzia et al., 2018). Furthermore, recommendations by the Australian Health Ministers’ Advisory Council (2012) state FV screening should take place at the first antenatal appointment. This means computerized screening may be particularly valuable in the context of antenatal care, where rapport may not yet be established with the midwife, and face-to-face disclosure may be challenging. Reducing the barrier of discomforts in making disclosures in new or unfamiliar interpersonal interactions may facilitate the screening process for the woman and clinician.

As Australia is a diverse country (Australian Bureau of Statistics, 2017), non-English speaking women comprise a growing proportion of those receiving antenatal care at Australian hospitals. In this study, midwives described language barriers as a hurdle to effective FV enquiry. Australian-based research exploring CALD women's experiences of accessing reproductive healthcare reflect this finding (Mengesha et al., 2016). According to Mengesha et al. (2016), women were at times unsure of the interpreter's ability to express their needs correctly to healthcare providers and often had a different interpreter at each visit. Given the challenges of screening non-English speaking women, midwives in this study emphasized the value of technology in allowing translation of the screening tool into the woman's own language. Translation of the screening tool may facilitate disclosure and help-seeking among women from non-English speaking backgrounds, which may be particularly valuable given these women may be less likely to seek help or report IPV (Ghafournia & Easteal, 2019). Australian-based research provides further justification for the integration of technology into IPV screening, with many CALD women indicating a preference for receiving IPV information electronically (Lee et al., 2013).

Health professional concern regarding computerized IPV screening has focused on how technology may interfere with the interpersonal element of patient-provider interactions, and become a barrier to communication (Bacchus et al., 2016; Decker et al., 2017; Scribano et al., 2011). The current study produced contrasting findings. Most midwives did not perceive technology as a barrier to communication, emphasizing the normality of technology use, and predicting that, regardless of modality, the woman will disengage from the midwife and engage with the screening tool. A willingness among midwives in the present study to incorporate technological screening tools may reflect changing societal acceptance of everyday technology use, and the increasing role of technology in healthcare, with technological innovation, such as the digitalization of medical records, driving important changes in the way care is provided (Cáceres, 2013; Gerrish et al., 2006).

In keeping with existing literature (Bacchus et al., 2016; Decker et al., 2017), midwives emphasized the importance of maintaining a personal presence during computer-based screening, so that technology does not become a replacement for human contact. In midwifery, empathy, openness and an awareness of the woman's feelings and thoughts are central to care (Bryar & Sinclair, 2011). This means technology must be embedded in a way that does not diminish the interpersonal nature of midwifery (Jacobson, 1993). In light of these findings, as well as previous research (Bacchus et al., 2016; Bryar & Sinclair, 2011; Chang et al., 2012; Choo et al., 2015; Decker et al., 2017), a positive way forward for IPV screening in antenatal care may involve a coupling of technological screening tools with face-to-face discussion.

### Strengths and Limitations

Sample adequacy relates to the appropriateness of the sample size and composition and is an important consideration in appraising the quality of qualitative research (Vasileiou et al., 2018). A sample size of 17 allowed data saturation (Sandelowski, 1995) to be achieved, with no new themes emerging in latter interviews, and the lack of gender diversity in the sample is representative of the female-dominated nature of midwifery. Furthermore, as most midwives were aware of the hospital's transition to EMR in future, this knowledge may have introduced biased responses if the midwives felt they should respond positively to the concept of computerized screening. Ten of the interviews were via telephone as this method of data collection was more convenient for some participants. Despite limitations such as a lack of visual cues to aid interpretation of speech and difficulty in achieving rapport (Robson, 1993), telephone interviews are thought to produce data comparable in quality to that attained by face-to-face interviews and have been associated with smaller interviewer effects and less tendency for socially desirable responses (Carr & Worth, 2001; Robson, 1993). This study may have limited transferability to rural settings such as satellite clinics, where connectivity and access to technology may be limited. Nevertheless, it provides a niche understanding of the views of midwives in the context of a metropolitan hospital in Australia.

## Conclusion

Midwives were receptive to computerized screening, highlighting how computerized modalities may contribute to overcoming limitations identified with paper-based screening. This study suggests that the implementation of a computerized FV screening modality which allows translation of the screening tool into different languages, provides prompts and guidance for the midwife, and educates and empowers the woman, would represent a positive advance in FV enquiry during antenatal care. Coupling technological screening tools with face-to-face discussion may better equip midwives to handle a disclosure and may enhance future women-centered, midwifery-led practices in family violence enquiry.

## Implications

Future recommendations for midwifery practice include continued training in FV enquiry, with midwifery champions, who have received more in-depth training, available to support and advise other midwives. Further research exploring how technological screening tools may be best designed and implemented to support midwives in their FV screening practices, promote quality of care, and empower women on pathways to safety is needed. Nevertheless, this study suggests that the implementation of a computerized screening modality which allows translation of the tool into different languages, provides prompts and guidance for the midwife, and educates and empowers the woman, would represent a positive advance in FV enquiry during antenatal care.

## Supplemental Material

sj-docx-1-vaw-10.1177_10778012251401898 - Supplemental material for Embedding Technological Tools Into Screening for Family Violence in Antenatal Care: Views of Australian Midwives Supplemental material, sj-docx-1-vaw-10.1177_10778012251401898 for Embedding Technological Tools Into Screening for Family Violence in Antenatal Care: Views of Australian Midwives by Minerva Kyei-Nimakoh, Caroline Bert and Kelsey Hegarty in Violence Against Women

## Data Availability

The data that support the findings of this study are available from the corresponding author, but restrictions apply to the availability of these data, which were used under license for the current study, and so are not publicly available. Data are, however, available from the corresponding author upon reasonable request.
