# Supplementary material for: Embedding Technological Tools Into Screening for Family Violence in Antenatal Care: Views of Australian Midwives
Source: Violence Against Women. 2025 Dec 12;32(15-16):5943–63. doi: 10.1177/10778012251401898 (PMC13672946; doi:10.1177/10778012251401898)
Supplement: Supplementary file 1 [file sj-docx-1-vaw-10.1177_10778012251401898.docx]

Consolidated criteria for reporting qualitative studies (COREQ): 32-item checklist

| **No** | **Item** | **Guide questions/description** | **Location in manuscript [section, page no.]** |
| --- | --- | --- | --- |
| **Domain 1: Research team and reflexivity** | | |  |
| Personal Characteristics | | |  |
| 1. | Interviewer/  facilitator | **Which author/s conducted the interview or focus group?**  All interviews were conducted by CB. | **Methods, page 4** |
| 2. | Credentials | **What were the researcher’s credentials?**  CB - BBMed  MKN – RN, BA, MPH, PhD  KH - MBBS OB/GYN RACGP PhD | - |
| 3. | Occupation | **What was their occupation at the time of the study?**  MKN - Research fellow and Honours student’s secondary supervisor.  CB - Honours student.  KH - Academic general practitioner, joint Chair in Family Violence Prevention at the University of Melbourne and the Royal Women's Hospital, leader of the NHMRC Safer Families Centre of Research Excellence, co-chair of the Melbourne Research Alliance to End Violence against Women (MAEVe) and Honours student’s primary supervisor. | - |
| 4. | Gender | **Was the researcher male or female?**  MKN - Female  CB - Female  KH - Female | - |
| 5. | Experience and training | **What experience or training did the researcher have?**  MKN - PhD completion in the field of nursing (maternal health).  CB - Novice researcher (completing Honours year)  KH - Extensive experience as an academic general practitioner and researcher in the field of family violence and domestic violence. | - |
| Relationship with participants | | |  |
| 6. | Relationship established | **Was a relationship established prior to study commencement?**  No | - |
| 7. | Participant knowledge of the interviewer | **What did the participants know about the researcher?**  CB - The participants understood that the research was part of CB’s Honours project.  MKN & KH - The participants knew that MKN and KH were CB’s supervisors and were researchers in the field of domestic and family violence. | - |
| 8. | Interviewer characteristics | **What characteristics were reported about the interviewer/facilitator?**  As this study was rooted in the context of technology playing an increasingly important role in the evolution of healthcare, in particular, with the introduction of electronic medical records (EMR) in the months following the study period, researchers utilised reflexivity to recognise any preconceptions regarding the value of technology in healthcare. | **Methods, page 5** |
| **Domain 2: Study design** | | |  |
| Theoretical framework | | |  |
| 9. | Methodological orientation and Theory | **What methodological orientation was stated to underpin the study?**  Thematic analysis utilising a combination of deductive and inductive approaches. | **Methods, page 5** |
| Participant selection | | |  |
| 10. | Sampling | **How were participants selected?**  Convenience and snowball sampling. | **Methods, page 4** |
| 11. | Method of approach | **How were participants approached?**  The provision of Expression of Interest forms at two midwifery team meetings, and snowball sampling via midwifery staff networks. | **Methods, page 4** |
| 12. | Sample size | **How many participants were in the study?**  Seventeen. | **Methods, page 4** |
| 13. | Non-participation | **How many people refused to participate or dropped out?**  Recruitment of participants was challenging at first, and repeated attempts at recruitment, via differing means, were required to obtain the number interviewed. However, there were no participants who subsequently refused to participate, withdrew consent or dropped out. | **Strengths and limitations, page 19** |
| Setting | | |  |
| 14. | Setting of data collection | **Where was the data collected?**  Seven face-to-face interviews were undertaken in private spaces at the hospital, and ten telephone interviews were conducted from a private meeting room at the Department of General Practice, University of Melbourne. | **Methods, page 4** |
| 15. | Presence of non-participants | **Was anyone else present besides the participants and researchers?**  No non-participants were present during data collection. | - |
| 16. | Description of sample | **What are the important characteristics of the sample?**  All participants were antenatal care midwives working at a large maternity hospital. Most were female (*n=16)* and were aged between 20-29 years (*n=6*), 30-39 years (*n=3*), 40-49 years (*n=6*) or 50-59 years *(n=2).* Despite diversity in years of practice, all had at least six months of experience as a midwife. Data was collected between April and August of 2019. | **Methods, page 4 & Results, page 6** |
| Data collection | | |  |
| 17. | Interview guide | **Were questions, prompts, guides provided by the authors? Was it pilot tested?**  Interviews were semi-structured and an interview guide was developed to facilitate discussion. Continued reflection on the transcripts allowed refining of the interview guide. Follow up questions were allowed. Examples of prompts are stated in the methods section of the manuscript. | **Methods, pages 4-5** |
| 18. | Repeat interviews | **Were repeat interviews carried out?**  No repeat interviews were carried out. | - |
| 19. | Audio/visual recording | **Did the research use audio or visual recording to collect the data?**  Interviews were audio recorded on two devices. | **Methods, page 5** |
| 20. | Field notes | **Were field notes made during and/or after the interview or focus group?**  Hand-written field notes were made during the interviews. These supplemented audio recordings and allowed documentation of general thoughts and observations. | **Methods, page 5** |
| 21. | Duration | **What was the duration of the interviews or focus group?**  Interviews had an average duration of 25 minutes. | **Methods, page 5** |
| 22. | Data saturation | **Was data saturation discussed?**  In the methods section, we state that continued reflection on the transcripts allowed refining of the interview guide and helped identify when data saturation was being approached. In the strengths and limitations section, we acknowledged that sampling continued until sufficiency was achieved. | **Methods, page 5 & Strengths and limitations, page 19** |
| 23. | Transcripts returned | **Were transcripts returned to participants for comment and/or correction?**  Transcripts were not returned to participants for comment or correction as they were anonymised at the time of transcription. | - |
| **Domain 3: analysis and findings** | | |  |
| 24. | Number of data coders | **How many data coders coded the data?**  All transcripts were coded by CB, however KH and MKN reviewed and validated the coding, and identified themes were discussed to reach a consensus, minimise individual bias and ensure validity. | **Methods, page 5-6** |
| 25. | Description of the coding tree | **Did authors provide a description of the coding tree?**  CB coded the data with handles that identified key concepts or reflections in an iterative manner, and an audit trail of code generation contributed to the rigour of the data analysis. Coding is described in the methods section of the manuscript. | **Methods, page 5** |
| 26. | Derivation of themes | **Were themes identified in advance or derived from the data?**  A combination of deductive and inductive approaches meant some themes were identified in advance, and others were identified from deeper exploration and interrogation of the data. | **Methods, page 5** |
| 27. | Software | **What software, if applicable, was used to manage the data?**  NVivo 12 software. | **Methods, page 5** |
| 28. | Participant checking | **Did participants provide feedback on the findings?**  No | - |
| Reporting | | |  |
| 29. | Quotations presented | **Were participant quotations presented to illustrate the themes / findings? Was each quotation identified?**  Yes, participant quotations were presented to illustrate each of the themes and findings. Each quotation was associated with an anonymised participant number. | **Results, pages 7-15** |
| 30. | Data and findings consistent | **Was there consistency between the data presented and the findings?**  Yes | - |
| 31. | Clarity of major themes | **Were major themes clearly presented in the findings?**  Yes, major themes are summarised and presented as bold sub-headings in the results section. | **Results, pages 7-15** |
| 32. | Clarity of minor themes | **Is there a description of diverse cases or discussion of minor themes?**  Minor themes and diverse cases are discussed within the results section of the manuscript. | **Results, pages 7-15** |
